# Supplementary material for: Valorization of Agricultural Waste as a Chemiresistor H2S-Gas Sensor: A Composite of Biodegradable-Electroactive Polyurethane-Urea and Activated-Carbon Composite Derived from Coconut-Shell Waste
Source: Polymers (Basel). 2023 Jan 29;15(3):685. doi: 10.3390/polym15030685 (PMC9920131; doi:10.3390/polym15030685)
Supplement: Supplementary file 1 [file polymers-15-00685-s001.zip › polymers-2163041-supplementary.pdf]

Supporting information

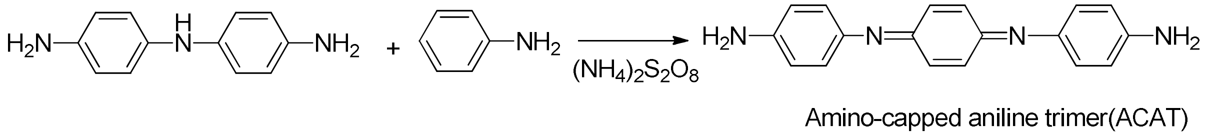

Figure S1: Preparation of amine-capped aniline trimer (ACAT)

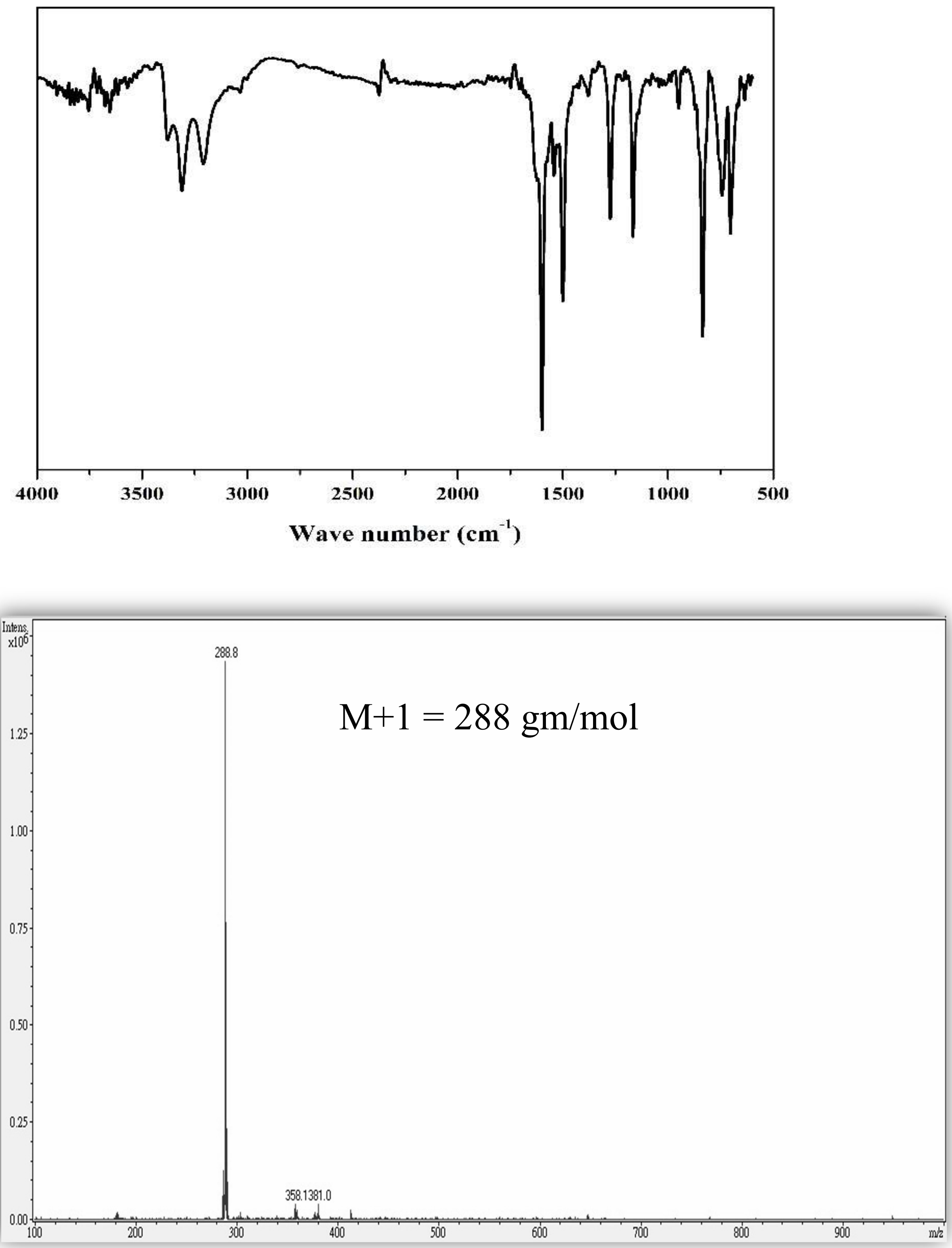

Figure S2. The representative FTIR spectrum and mass spectrum of ACAT.

Response time

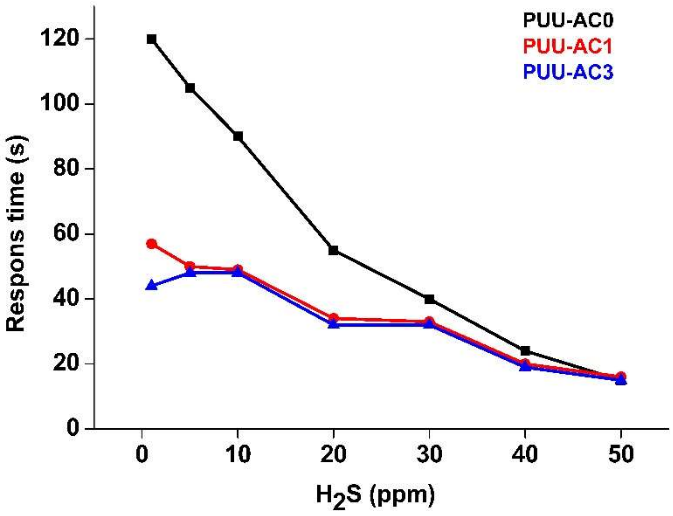

Recovery time

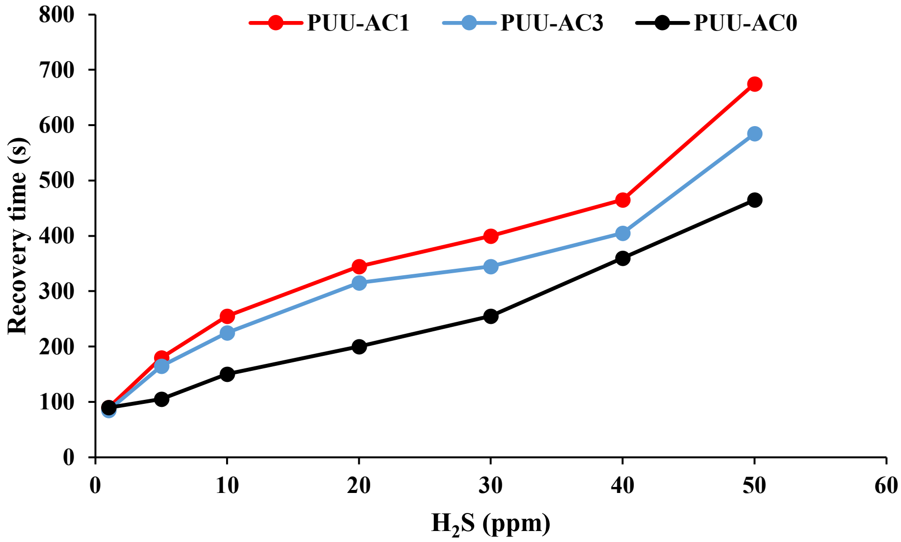

Figure S3 Response time (a) and recovery time of PUU-AC0, PUU-AC1 and PUU-AC3 sensors towards H_2S gas concentration ranging from 1-50 ppm at room temperature.
